# Generalized Self-Efficacy, Dispositional Optimism, and Illness Acceptance in Women with Polycystic Ovary Syndrome

**DOI:** 10.3390/ijerph15112484

**Published:** 2018-11-07

**Authors:** Ewa Rzońca, Grażyna Iwanowicz-Palus, Agnieszka Bień, Artur Wdowiak, Ryszard Szymański, Gustaw Chołubek

**Affiliations:** 1 Faculty of Health Sciences, Medical University of Lublin, 4-6 Staszica St., 20-081 Lublin, Poland; eva.rzonca@gmail.com (E.R.); spupalus@gmail.com (G.I.-P.); 2 Diagnostic Techniques Unit, Medical University of Lublin, 4-6 Staszica St., 20-081 Lublin, Poland; wdowiakartur@gmail.com (A.W.); gustaw.cholubek@umlub.pl (G.C.); 3 Gynecological-obstetrics Ward, Independent Public Complex of Health Care Facilities in Nowa Dęba, 1a M.C. Skłodowska St., 39-460 Nowa Dęba, Poland; rsz18@poczta.fm

**Keywords:** polycystic ovary syndrome, self-efficacy, optimism, acceptance of illness

## Abstract

Polycystic ovary syndrome (PCOS) is one of the most common chronic endocrinopathies affecting between 5 and 10% of reproductive age women. A diagnosis of PCOS very often causes a deterioration in the woman’s self-esteem and self-image, and consequently her quality of life (QoL). The purpose of the study was to investigate generalized self-efficacy, dispositional optimism and acceptance of illness in women with PCOS and to explore factors that affect these variables. The study was performed between January and November 2016 among women with PCOS using health care services. The study used a diagnostic survey with questionnaires. The research instruments included the Generalized Self-Efficacy Scale (GSES), the Life Orientation Test-Revised (LOT-R), the Acceptance of Illness Scale (AIS), and a standardized interview questionnaire. Among the PCOS patients studied, the mean score for generalized self-efficacy was 28.74 (±5.16), dispositional optimism—13.56 (±4.28), and acceptance of illness—27.90 (±7.74). The respondents’ generalized self-efficacy was determined by their residence, education, socio-economic standing, BMI, and time from diagnosis (*p* < 0.05), while socio-economic standing was a determinant of dispositional optimism (*p* < 0.05). Determinants of illness acceptance in women with PCOS included their residence, socio-economic standing, and time from diagnosis (*p* < 0.05). Increased generalized self-efficacy and dispositional optimism contributed to more illness acceptance in PCOS patients. The present study, compared with others on the subject, will enable specialists providing care to women with PCOS to gain a deeper and more comprehensive understanding of the situation and condition of their patients. It will also allow for a better response to the needs of PCOS patients, and provide them with individualized, holistic specialist care, diagnostics, and treatment.

## 1. Introduction

Every person reacts differently to the diagnosis of a chronic illness, in accordance with their individual characteristics. Any illness affects the psychological state and functioning of the person diagnosed with it, and chronic illnesses are very often associated with a number of adaptations and changes in a person’s life and activities [1,2,3]. Significant factors affecting the acceptance of illness include a person’s belief in their capacity to adapt, their self-esteem, self-efficacy, optimism, and socio-demographic factors [1,2,3,4].

Polycystic ovary syndrome (PCOS) is one of the most common chronic endocrinopathies affecting between 5 and 10% of reproductive age women. The syndrome presents a variety of symptoms, including hyperandrogenism, menstrual disorders, obesity, metabolic syndrome, type 2 diabetes mellitus, fertility disorders, or emotional and psychological disorders, while its etiology has not yet been fully understood [5,6,7]. Polycystic ovary syndrome is most commonly diagnosed based on the Rotterdam criteria, i.e., when two out of the following three findings are present: oligomenorrhea or oligo-ovulation, clinical and/or biochemical hyperandrogenism, and/or polycystic ovaries on ultrasound [7]. A diagnosis of PCOS very often causes a deterioration in the woman’s self-esteem and self-image, and consequently her quality of life (QoL) [8,9]. Polycystic ovary syndrome presents a broad variety of symptoms, including metabolic, endocrine, and emotional or psychological ones, which is extremely challenging to women diagnosed with the disorder [5,7,10]. Numerous studies on PCOS focus on its biological and physiological aspects, though a growing number of publications discuss the adverse impact of PCOS on women’s lives and how women function. These publications emphasize the fact that PCOS patients are at a higher risk of depression, anxiety, and low QoL [5,6,7,8,9,10,11,12,13]. Thus, women with PCOS, faced with the challenge of such a multidimensional disorder, must cope with their new situation. This may be affected by multiple factors, including the patient’s individual predispositions [6,8,9,10,11,12,13].

Individual capabilities and predispositions, including optimism or self-efficacy, have an impact on a person’s acceptance of their situation, including the situation of being ill, and this motivated the authors to tackle this subject in the present paper. The purpose of the study was to investigate generalized self-efficacy, dispositional optimism and acceptance of illness in women with PCOS and to explore factors that affect these variables.

## 2. Materials and Methods

### 2.1. Participants

The cross-sectional study was performed between January and November 2016 in PCOS patients using health care services (primary care, specialist outpatient care, and inpatient/hospital care) in four regions of Poland: Lubelskie, Podkarpackie, Pomorskie, and Wielkopolskie provinces. Inclusion criteria for cases were as follows: Age over 18 years, PCOS diagnosis made based on the Rotterdam criteria, and use of health care services in Poland. Exclusion criteria were: Cancer and psychological disorders. The study was performed in accordance with the Helsinki Declaration, and approved by the Lublin Medical University Bioethics Committee (approval no. KE-0254/189/2015). It was also approved by the respective health care units. Respondents were informed that participation was voluntary, and that study results were anonymous and to be used exclusively for research purposes. Out of the 300 survey questionnaires distributed to respondents, 250 correctly completed questionnaires were analyzed. Fifty questionnaires were excluded from further analysis due to incomplete data. The data effectiveness rate was 83.33%.

### 2.2. Assessments

The study used a diagnostic survey with questionnaires. Research instruments included the Generalized Self-Efficacy Scale (GSES), the Life Orientation Test-Revised (LOT-R), the Acceptance of Illness Scale (AIS), and an own sociodemographic questionnaire to collect data on respondents’ characteristics (age, residence, marital status, education, professional activity, socio-economic standing—self-reported, having children, BMI—collected from respondents’ medical records, time from PCOS diagnosis).

The GSES was developed by Schwarzer and Jerusalem, and adapted for Polish settings by Juczyński. It measures an individual’s sense of generalized self-efficacy in difficult situations. The scale comprises 10 statements, which are scored by the respondent as follows: 1—not true at all, 2—hardly true, 3—moderately true, 4—exactly true. The total score ranges between 10 and 40 points, representing a person’s level of self-efficacy. Higher scores indicate a stronger sense of self-efficacy. Scores should be interpreted using sten ranges, with scores of sten 1–4 considered low, and sten 7–10 considered high. The scale has good psychometric properties, with a Cronbach’s α of 0.85 [14].

The LOT-R was authored by Scheier, Carver, and Bridges, and adapted for Polish settings by Poprawa and Juczyński. It measures dispositional optimism based on 10 statements rated 0–4, where 0 stands for “I disagree a lot”, while 4 stands for “I agree a lot”. Total score ranges between 0 and 24 points, with higher scores denoting more optimism. The raw score is converted into sten results, with scores of sten 1–4 considered low, i.e., indicating pessimistic tendencies, while sten 7–10 scores are considered high, i.e., indicating an optimistic attitude. The reliability of the LOT-R as measured by Cronbach’s α is 0.76 [15].

The AIS was developed by Felton et al., and adapted for Polish settings by Juczyński. It allows for measuring illness acceptance in adults, using 8 statements concerning the negative consequences of poor health, rated by the respondents using a 5-item scale, with 1 standing for “strongly agree”, and 5 for “strongly disagree”. The total, between 8 and 40 points, is a measure of illness acceptance. Low scores indicate a lack of acceptance, poor adaptation to the illness, and significant emotional problems associated with the illness, while high scores indicate a respondent’s acceptance of their condition. Internal consistency as measured by Cronbach’s α is 0.85 for the original version of the AIS, and very similar for the Polish version, with an α of 0.82 [16].

### 2.3. Statistical Analyses

Statistical analysis of data from the questionnaires was performed using STATISTICA version 12 (StatSoft, Kraków, Poland). Qualitative data are described by numbers (n) and percentages (%), and quantitative data are described by means (M), standard deviations (SD) and medians (Me). To analyze the variables affecting GSES, LOT-R, and AIS scores, standard multiple regression analysis was performed. With this method, results are interpreted by comparing *β* values, indicating the direction and strength of relationships between predictors. The fit of the regression model to the data is indicated by the corrected *R*^2^ value. Reference categories were provided for nominal and ordinal variables: For age it was under 25 years old, for residence—urban: Province capital, for education—college/university, and for time from PCOS diagnosis—less than 1 year. As to BMI, it was included in the analysis as a scale variable. If a variable comprised two categories, the analysis involved comparing the two categories, e.g., in the case of marital status—single, professional activity—not working, socio-economic status—unsatisfactory, and for having children—none. Correlations between selected variables were tested using Pearson’s r; correlation strength was evaluated using Guilford’s classification. The study used a significance threshold of *p* < 0.05.

## 3. Results

The study included 250 women with PCOS. Most respondents were women aged 26–35 years (43.20%), lived in urban: Province capital (38.40%), married (64.40%), college/university-educated (59.60%), professionally active (77.20%), who viewed their socio-economic standing as satisfactory (62.40%), had no children (53.20%), mean BMI 26.41, and had been diagnosed with PCOS 1 to 5 years before (50.40%) (Table 1).

Table 2 shows the mean scores for generalized self-efficacy (28.74 ± 5.16), dispositional optimism (13.56 ± 4.28), and acceptance of illness (27.90 ± 7.74).

The regression model for variables generalized self-efficacy (GSES) is shown in Table 3. The regression model that was developed accounts for 18.8% of variance for the GSES variable (*F*(15.234) = 4.834; *p* < 0.001). Statistically significant predictors for the GSES regression model included residence—urban: Other (*β* = −0.140; *p* = 0.033), education—primary/vocational (*β* = −0.137; *p* = 0.033), socio-economic standing (*β* = 0.275; *p* = 0.001), BMI (*β* = −0.152; *p* = 0.012) and time from PCOS diagnosis—1–5 years (*β* = −0.233; *p* = 0.010) and 6–10 years (*β* = −0.197; *p* = 0.023). Living in a city other than a province capital, having finished one’s education at the primary/vocational level, having a higher BMI, and a 1 to 10 years from PCOS diagnosis (1–5 and 6–10 years) were all associated with a lower sense of self-efficacy in the women studied. Better socio-economic status predicted a stronger sense of self-efficacy.

Table 4 shows regression model for variables LOT-R. The model was found to fit the data well (*F*(15.234) = 2.427; *p* = 0.003), while it only accounted for 7.9% of variance in the LOT-R variable. For the LOT-R variable, the only statistically significant variable was socio-economic standing (*β* = 0.259; *p* = 0.001)—the better the socio-economic status, the higher the optimism score.

The regression model for AIS is shown in Table 5. This regression model accounts for 20.7% of variance for the AIS variable (*F*(115.234) = 5.340; *p* < 0.001). For the AIS variable model, statistically significant predictors included residence—urban: Other (*β* = −0.134; *p* = 0.037), socio-economic standing (*β* = 0.335; *p* = 0.001), and time from PCOS diagnosis—1–5 years (*β* = −0.263; *p* = 0.003). Living in a city other than a province capital and time from PCOS diagnosis between 1 and 5 years were associated with poorer acceptance of illness by the PCOS patients studied, while better socio-economic standing predicted more PCOS acceptance.

Statistical analyses showed significant positive correlations between generalized self-efficacy on the one hand, and dispositional optimism and illness acceptance on the other, as well as between dispositional optimism and illness acceptance (*p* < 0.001). The strength of correlations was rated between 0.434 and 0.610 (Table 6).

## 4. Discussion

Illness, especially a chronic one such as diabetes, cardiovascular disease, or thyroid disease, is a major challenge for the person diagnosed with it. It has a considerable impact on the patient’s life and functioning, which is associated with multiple and diverse factors, including concern about one’s health and life, possible complications, impact of the disease on family or professional life, self-efficacy, optimism, and a number of other social, demographic, and economic considerations [2,10,17,18,19]. Polycystic ovary syndrome matches the above description of the impact illness has on the patient’s life, as the literature emphasizes that a diagnosis of PCOS affects the whole of a woman’s life. Symptoms of PCOS first manifest during puberty or in early adulthood, but its complications or sequelae have a negative impact on women’s functioning and QoL at subsequent stages as well, including menopause or even old age [5,7,10,20,21,22,23]. Therefore, the authors of the present paper attempted to evaluate generalized self-efficacy, dispositional optimism, and illness acceptance in women with PCOS, as well as the determinants of these characteristics.

Self-efficacy is strongly associated with an ability to control one’s actions so as to achieve the desired results, even in unfavorable circumstances. It reflects a person’s beliefs about their capability to change their behavior when diagnosed with an illness or to prevent potential health problems [24,25,26]. The author of the Polish adaptation of GSES reported mean scores for Polish populations, including a population of women who had undergone a mastectomy—30.07 [14]. In Polish hysterectomy patients, a self-efficacy score of 32.46 was reported [1], in University of the Third Age students—a mean of 30.12 [25], and in patients with heart failure—32 [27]. These data indicate a strong sense of self-efficacy in the various groups [1,14,25,27]. As to the present study, it demonstrated a moderate level of self-efficacy in PCOS patients (28.74), similar to that found in diabetics (28.34) or in women with migraines (28.57) [14].

Kozica et al. (2013) studied self-efficacy and self-care in women with and without PCOS, demonstrating that significant predictors of self-efficacy in their respondents included health vigilance, overall health history, and diagnosed infertility. The authors state that women with PCOS should be aware that their illness is a chronic condition that requires them to carefully manage their health by introducing lifestyle modifications and undergoing regular screening [24]. Rogala et al. (2015) investigated the relationship between mental adaptation to cancer and self-efficacy in women who had undergone hysterectomies due to cancer. Their analysis included socio-demographic factors, such as education, residence, relationship status, and economic standing, which could affect the respondents’ self-efficacy. The only statistically significant variable was the patients’ relationship status, as women in a relationship had a higher level of self-efficacy than single ones [1]. In a study by Zielińska-Więczkowska (2017), concerning the relationships between satisfaction with life and selected personal resources in University of the Third Age students, self-efficacy was associated with education, financial standing, and perceived health. Lower levels of education were associated with poorer self-efficacy, while a better financial standing or better perceived health was related to higher GSES scores [25]. In the present study, lower levels of self-efficacy were found in PCOS patients who lived in cities other than province capitals, had had a primary/vocational education, had higher BMI values, and had been living with PCOS for a longer time (1–10 years), while higher levels were found in women with a better socio-economic standing.

The present study also analyzed another important individual characteristic or tendency, namely optimism, defined as an overall expectation of positive rather than negative life experiences in the future. This trait is conducive to motivation, perseverance, and determination in pursuing and achieving goals [2,28,29,30]. The literature on the subject provides a highly inconsistent view on dispositional optimism and its predictors in various patient groups studied in the world, depending on their health status [2,25,28,29,30,31]. In their study on the relationship between dispositional optimism and acceptance of illness in a group of patients with Graves’ disease, Basińska et al. (2008) found comorbidity to be the only variable significantly affecting the patients’ optimism levels. Those diagnosed with other diseases beside Graves’ had lower levels of dispositional optimism than those with no comorbidities [2]. A study by Chung et al. (2016) showed that, in stroke patients, there was a negative correlation between optimism and depression symptoms—lower optimism levels were associated with more severe symptoms of depression [28]. Bleil et al. (2012), in turn, found that a more pessimistic attitude may contribute to the failure of fertility treatment [29]. Moyer et al. (2009) analyzed optimism/pessimism and health-related quality of life (HRQoL) in pregnant women from China, Ghana, and the US. Optimism levels were found to be associated with the women’s country of origin, professional activity, education, and treatment for emotional disorders [30]. Zielińska-Więczkowska (2017) demonstrated that financial standing had a significant impact on dispositional optimism in the University of the Third Age students she studied [25]. The present results corroborate those reported by this author. Satisfaction with one’s socio-economic standing was associated with a higher level of dispositional optimism, which overall was moderate in the PCOS patients studied.

Acceptance of illness is a very important stage in the life of a person diagnosed with a disease, especially a chronic one. It allows the patient to adapt to their new situation, and higher levels of illness acceptance contribute to better coping with the illness, and ultimately, to a better QoL [2,32,33]. In the study on Graves’ patients by Basińska et al. (2008), the reported acceptance of illness score was 28.48 [2]. In turn, Bień et al. (2016) reported a score of 30.66 in a group of patients with gestational diabetes mellitus (GDM) [32]. A comparison between the cited scores and the mean score of 27.90 found in the present study demonstrates that PCOS patients have a lower level of illness acceptance than other populations. This finding may be explained by the complexity and variety of PCOS symptoms, which significantly affect the life of patients, as well as their self-perception in relation to the expectations of the contemporary world [6,8,9,24,34].

The multiple factors affecting acceptance of illness also have an impact on a person’s adaptation to the illness, which occurs to various degrees, as shown in the literature on the subject, as well as in another part of the present study [2,32,33,35]. In a study by Jankowska-Polańska et al. (2016), determinants of illness acceptance in patients with chronic obstructive pulmonary disease (COPD) included age, education, and duration of illness. Better illness acceptance was found in younger COPD patients, in those with college/university education, and with a shorter duration of illness [35]. Bień et al. (2016) demonstrated that acceptance of illness in women with GDM improved with very good financial standing, dietary treatment of diabetes, and very good overall perceived health [32]. In turn, Kostyła et al. (2013), in a study on patients with psoriasis, found acceptance of illness to increase as psycho-pathological symptom intensity decreased [33]. In the present study, PCOS patients who lived in cities other than province capitals and had been diagnosed with PCOS between 1 and 5 years before were found to have lower levels of illness acceptance. Better socio-economic standing was associated with greater acceptance of PCOS in the group studied.

The findings of the present study demonstrate a single common determinant of all the aspects analyzed, including self-efficacy, dispositional optimism, and acceptance of illness: The PCOS patients’ socio-economic standing. The literature on the subject emphasizes the importance of financial situation for health, as it affects access to health care in general and to specific methods of treatment, the ability to maintain a healthy lifestyle, as well as how the patients manage on a daily basis and their family life [19,25,32,36].

The final stage of the present study involved an analysis of correlations between self-efficacy, dispositional optimism, and acceptance of illness in women with PCOS. Popa-Velea & Purcarea (2014), investigating the psychological factors affecting HRQoL in COPD patients, demonstrated that self-efficacy and optimism were positively correlated with the patients’ HRQoL. Moreover, patients with low levels of self-efficacy and optimism may experience more problems and discomfort than those with higher levels of these traits in the case of a comparable decrease in lung function [37]. Zielińska-Więczkowska (2017) found satisfaction with life to be positively correlated both with generalized self-efficacy and with dispositional optimism [25]. Basińska et al. (2008) demonstrated an association between dispositional optimism and acceptance of illness in their group of patients with Graves’ disease [2]. As to the present study, a stronger sense of self-efficacy was associated with both greater optimism and more acceptance of PCOS, and similarly, higher levels of optimism were associated with greater illness acceptance. This warrants the conclusion that acceptance of illness is the product of an individual’s personal resources that allow them to come to terms with their illness and to adapt to it, so as to live and function better with the illness, as evidenced both by literature reports and by the present study [2,25,37].

So far, the authors have found no studies on the level and determinants of generalized self-efficacy, dispositional optimism, and illness acceptance among women with PCOS in the available literature. Therefore, the study is an original one. The present study has certain limitations. The study concept did not include an analysis of the impact of PCOS symptoms (including hirsutism or menstrual disorders) or psychological condition on the patient’s life, though these analyses were included in a number of studies worldwide, providing information and direction to the authors of the present paper. Further studies are still warranted on PCOS, its clinical circumstances, etiology, impact on patients’ lives, and long-term consequences.

## 5. Conclusions

Women with PCOS have moderate levels of generalized self-efficacy, dispositional optimism, and illness acceptance. The patients’ generalized self-efficacy is determined by their residence, education, socio-economic standing, BMI, and time from diagnosis, while socio-economic standing was the only determinant of dispositional optimism. Determinants of illness acceptance in women with PCOS included their residence, socio-economic standing, and time from diagnosis. Increased generalized self-efficacy and dispositional optimism contributed to greater illness acceptance in PCOS patients.

The present study, compared with others on the subject, will enable specialists providing care to women with PCOS to gain a deeper and more comprehensive understanding of the situation and condition of their patients. It will also allow for a better response to the needs of PCOS patients, and provide them with individualized, holistic specialist care, diagnostics, and treatment.

## Figures and Tables

**Table 1:** Participants’ characteristics.

Participants’ Characteristics		*n*	%
Age	Under 25 years old	47	18.80
	26–35	108	43.20
	Over 35 years old	95	38.00
Residence	Urban: Province capital	96	38.40
	Urban: Other	74	29.60
	Rural	80	32.00
Marital status	Single	89	35.60
	Married	161	64.40
Education	Primary/vocational	22	8.80
	High school	79	31.60
	College/university	149	59.60
Professional activity	Working professionally	193	77.20
	Not working	57	22.80
Socio-economic standing	Unsatisfactory	94	37.60
	Satisfactory	156	62.40
Having children	No children	133	53.20
	Children	117	44.80
Body Mass Index (BMI)	Mean BMI	26.41	
Time from PCOS diagnosis	Up to 1 year	38	15.20
	1–5 years	126	50.40
	6–10 years	52	20.80
	more than 10 years	34	13.60

**Table 2:** Mean scores for generalized self-efficacy (GSES), dispositional optimism (LOT-R), and illness acceptance (AIS) in the polycystic ovary syndrome (PCOS) patients studied.

	M	Me	SD
**GSES**	28.74	30	5.16
**LOT-R**	13.56	13	4.28
**AIS**	27.90	28	7.74

M—mean; SD—standard deviation; Me—median.

**Table 3:** Regression model for variables GSES.

Variables	GSES *R*^2^ = 0.188 *F*(15.234) = 4.834 *p* < 0.001			
	*B*	*β*	*t*	*p*
Age: 26–35	0.023	0.002	0.024	0.981
Age: Over 35 y/o	−0.090	−0.008	−0.076	0.939
Residence: Urban—other	−1.576	−0.140	−2.150	0.033
Residence: Rural	−1.216	−0.110	−1.665	0.097
Marital status	0.315	0.029	0.438	0.662
Education: Primary/vocational	−2.487	−0.137	−2.148	0.033
Education: High school	−0.001	0.000	−0.002	0.999
Professional activity	−0.676	−0.055	−0.904	0.367
Socio-economic standing	2.921	0.275	4.561	0.001
Having children	0.103	0.010	0.127	0.899
BMI	−0.169	−0.152	−2.541	0.012
Time from PCOS diagnosis: 1–5 years	−2.395	−0.233	−2.599	0.010
Time from PCOS diagnosis: 6–10 years	−2.504	−0.197	−2.282	0.023
Time from PCOS diagnosis: More than 10 years	0.319	0.021	0.246	0.806

**Table 4:** Regression model for variables LOT-R.

Variables	LOT-R *R^2^* = 0.079 *F*(15.234) = 2.427 *p* = 0.003			
	*B*	*β*	*t*	*p*
Age: 26–35	0.009	0.001	0.010	0.992
Age: Over 35 y/o	0.016	0.002	0.015	0.988
Residence: Urban—other	−0.851	−0.091	−1.313	0.190
Residence: Rural	−0.572	−0.062	−0.886	0.377
Marital status	−0.192	−0.021	−0.303	0.762
Education: Primary/vocational	−1.506	−0.100	−1.472	0.142
Education: High school	−0.167	−0.018	−0.271	0.787
Professional activity	−0.855	−0.084	−1.293	0.197
Socio-economic standing	2.284	0.259	4.034	0.001
Having children	0.489	0.057	0.691	0.490
BMI	−0.040	−0.044	−0.688	0.492
Time from PCOS diagnosis: 1–5 years	1.253	0.147	1.538	0.125
Time from PCOS diagnosis: 6–10 years	1.324	0.126	1.366	0.173
Time from PCOS diagnosis: More than 10 years	2.025	0.162	1.763	0.079

**Table 5:** Regression model for variables AIS.

Variables	AIS *R^2^* = 0.207 *F*(15.234) = 5.340 *p* < 0.001			
	*B*	*β*	*t*	*p*
Age: 26–35	1.292	0.083	0.885	0.377
Age: Over 35 y/o	−1.914	−0.120	−1.096	0.274
Residence: Urban—other	−2.272	−0.134	−2.092	0.037
Residence: Rural	−1.918	−0.116	−1.772	0.078
Marital status	1.722	0.107	1.617	0.107
Education: Primary/vocational	−3.145	−0.115	−1.834	0.068
Education: High school	1.347	0.081	1.302	0.194
Professional activity	−0.168	−0.009	−0.152	0.880
Socio-economic standing	5.335	0.335	5.623	0.001
Having children	0.758	0.049	0.628	0.530
BMI	−0.077	−0.046	−0.777	0.438
Time from PCOS diagnosis: 1–5 years	−4.056	−0.263	−2.971	0.003
Time from PCOS diagnosis: 6–10 years	−2.169	−0.114	−1.334	0.183
Time from PCOS diagnosis: More than 10 years	−0.016	0.000	−0.009	0.993

**Table 6:** Correlations between GSES, LOT-R, and AIS in the PCOS patients studied.

	GSES	LOT-R	AIS
**GSES**	–	*r* = 0.474	*r* = 0.610
	–	*p* < 0.001	*p* < 0.001
**LOT-R**	*r* = 0.474	–	*r* = 0.434
	*p* < 0.001	–	*p* < 0.001
**AIS**	*r* = 0.610	*r* = 0.434	–
	*p* < 0.001	*p* < 0.001	–

GSES—generalized self-efficacy; LOT-R—dispositional optimism; AIS—illness acceptance.
